# High-Density Lipoprotein Cholesterol in Age-Related Ocular Diseases

**DOI:** 10.3390/biom10040645

**Published:** 2020-04-22

**Authors:** Bjorn Kaijun Betzler, Tyler Hyungtaek Rim, Charumathi Sabanayagam, Chui Ming Gemmy Cheung, Ching-Yu Cheng

**Affiliations:** 1 Yong Loo Lin School of Medicine, National University of Singapore, Singapore 119228, Singapore; bjorn.betzler@u.nus.edu; 2 Singapore Eye Research Institute, Singapore National Eye Centre, Singapore 169856, Singapore; tyler.rim@snec.com.sg (T.H.R.); charumathi.sabanayagam@seri.com.sg (C.S.); gemmy.cheung.c.m@singhealth.com.sg (C.M.G.C.); 3 Ophthalmology & Visual Sciences Academic Clinical Program (EYE-ACP), Duke-NUS Medical School, Singapore 169857, Singapore; 4 Department of Ophthalmology, Yong Loo Lin School of Medicine, National University of Singapore, Singapore 119228, Singapore

**Keywords:** age-related macular degeneration, cataract, diabetic retinopathy, macular edema, glaucoma, intraocular pressure, visual impairment, ageing, high-density lipoprotein, dyslipidaemia

## Abstract

There is limited understanding of the specific role of high-density lipoprotein cholesterol (HDL-C) in the development of various age-related ocular diseases, despite it being a common measurable biomarker in lipid profiles. This literature review summarizes current knowledge of the role of HDL-C, if any, in pathogenesis and progression of four age-related ocular diseases, namely age-related macular degeneration (AMD), age-related cataract, glaucoma, and diabetic retinopathy (DR), and will primarily discuss epidemiological and genetic evidence.

## 1. Introduction

High density lipoprotein (HDL) is a set of biomolecules, playing diverse metabolic, regulatory and homeostatic roles in the human body. HDL particles have a heterogeneous biochemical composition, accompanied by distinct physiological and biochemical properties that can be altered in several disease states, pathological conditions, by genetic variation, and through controllable diet and lifestyle changes [1,2,3,4,5,6,7]. One of these components is cholesterol, and we, thus, refer to the cholesterol portion carried within HDL particles as high-density lipoprotein cholesterol (HDL-C) in this manuscript. Despite the ever-increasing knowledge of HDL-C, the precise role of HDL-C in age-related ocular diseases is not fully understood. Most current literature focuses on the associations between ocular diseases and the broader category of dyslipidaemias. With a growing ageing population, age-related ocular diseases are increasingly commonplace. Recent studies have reported a global prevalence of 33.4% for diabetic retinopathy (DR) (≥ 50 years) [8], 3.54% for primary open-angle glaucoma (aged 40–80 years) [9] and 8.69% for any age-related macular degeneration (AMD) (aged 45 years or older), and the prevalences increased dramatically with age [10]. Untreated cataracts are the leading cause of blindness in those aged 50 years or older in 2015 [11]. These conditions are accompanied by substantial socioeconomic burden, and there is an urgency to find new treatment strategies that could prevent or delay their onset or progression. This review streamlines current knowledge of HDL & its HDL-C subset in relation to four common ophthalmological conditions—AMD, age-related cataract, glaucoma and DR. It covers information ranging from epidemiology, pathophysiology to therapeutics, with an emphasis on the role of HDL-C in humans. Such knowledge may help understanding the basis of diseases and developing new and improved treatment strategies targeting HDL or HDL-C levels.

To identify relevant literature, a database search with the key words “age-related macular degeneration”, “aging”, “cataract”, “retinopathy”, “macular edema”, “glaucoma”, “Intraocular pressure”, “high-density lipoprotein” and “lipid” was conducted. Search results were screened for relevance. References cited within the identified articles were used to further augment the search. This review encompassed an international search, but only articles published in English have been used. There was no time limit for study inclusion.

## 2. HDL-C in Age-Related Macular Degeneration

### 2.1. Background of AMD

Age-related macular degeneration (AMD) is a leading cause of irreversible vision loss in the elderly in western countries [12,13]. Population ageing is expected to further increase the prevalence of AMD cases; 8.7% of the global population has AMD, and the absolute number of cases is projected to climb to approximately 196 million in 2020, and to 288 million in 2040 [10]. This will place a significant burden on healthcare systems and increase its economic burden [14,15]. AMD is etiologically complex, with many environmental, behavioural, and genetic factors influencing the risk of disease onset and development [12,16,17]. While drusen formation is primarily an age-related phenomenon, it predisposes an individual to develop AMD. Drusen are focal accumulations of proteins, lipids and minerals, deposited at the interface between the retinal pigment epithelium (RPE) basal lamina and Bruch’s membrane [18,19,20,21]. As AMD progresses, drusen enlarge and become more numerous, often accompanied by signs of pigmentary abnormalities [22], and photoreceptor cell degeneration at the macula. As AMD develops, it manifests as a small dark spot at the centre of one’s visual field that gets progressively larger over time, impairing the patient’s central vision—crucial for activities such as reading and facial recognition. Progression to late AMD has two distinct clinical end-stages—Geographic Atrophy (GA) and Neovascular AMD. GA leads to a slower but irreversible degeneration of the RPE and photoreceptor cells. It is non-neovascular. Neovascular AMD is characterized by abnormal infiltration of fragile, new blood vessels growing. Anti-Vascular endothelial growth factor (VEGF) therapies have been proven to be a viable therapeutic option for neovascular AMD.

### 2.2. Epidemiological Evidence

It is established that elevated HDL-C supports reverse cholesterol transport and improves endothelial function, which decreases the risk of atherosclerosis [23]. However, epidemiological studies evaluating the role of HDL-C levels in AMD have yielded varying, and often conflicting findings on the association between HDL-C and AMD (Table 1). In 2018, Kersten et al. reviewed 55 studies that measured HDL-C levels in AMD [24]. While one might expect an inverse association between HDL-C levels and AMD, the majority (34 studies) showed no association; 16 studies showed higher HDL-C levels in AMD; five studies showed lower HDL-C levels in AMD compared to controls. A 2016 meta-analysis showed that AMD incidence increased by approximately 18% for an increment of 1 mmol/L. (Relative risk (RR) = 1.18; 95% CI = 1.01–1.35; *p*-value = 0.007) [25]. Mendelian randomization studies using genetic variants associated with lipid fractions also support the hypothesis that high HDL-C is a causal risk factor for AMD [26], with an odds ratio (OR) estimate of 1.22 (95% CI 1.03–1.44) per 1 standard deviation increase in HDL-C. With regard to HDL particle size, Cheung et al. [27] established increased risk of neovascular AMD in individuals with increased serum HDL concentration, driven by an excess of medium-sized particles (8.2–8.8 nm). It has also been suggested that elderly patients with elevated serum HDL are more susceptible to developing AMD [28]. The Alienor Study involved 963 individuals with an average age of 80.2 years; higher HDL-C was significantly associated with an increased incidence of early (OR = 2.45, 95% CI = 1.54–3.90; *p*-value = 0.0002) and any AMD (OR = 2.29, 95%CI = 1.46–3.59; *p*-value = 0.0003). On the other hand, inverse correlations between HDL-C levels and AMD have also been reported. Increasing HDL-C was inversely related to incident late AMD in the Blue Mountain Eye Study [29]. A 2010 cross-sectional study reported that odds of early AMD decreased by approximately 10% per 5 mg/dL (0.13 mmol/L) increase in HDL level. (OR = 0.91; 95% CI = 0.83-0.998) [30].

Looking at these results collectively, we cannot conclude that HDL-C levels are positively correlated with increased risk of AMD. While studies reflecting either a positive or negative association are available, and there are slightly more studies reporting a positive association, the majority of studies are still ones that report no significant association. Therefore, further clarification and evidence of this link will be beneficial. Such insights could potentially lead to a clearer view when HDL-C measurements are involved in considering patient management. However, the pathogenic mechanisms, whether direct or indirect, are largely unknown and full of gaps. This will be addressed in the next section. The reasons for inconsistencies across studies are not understood, but differences in how studies defined AMD end points, study sample characteristics including sex and age distribution, and changing patterns of medication may contribute to this lack of consistency. Long-term follow-up studies are needed to understand the impact of HDL-C on AMD risk and progression over time.

### 2.3. Laboratory Evidence

Current literature largely proposes that there is some form of a pathological mechanism between HDL-C and AMD. It is yet unclear whether direct or indirect causation is involved, or whether the net correlation is positive or inverse. While it is likely that more than one biological mechanism/pathway is involved, more research into the relative strength of each mechanism, how they interact together, and the effect by different variables such as age, gender and ethnicity will be beneficial. Studies on HDL, AMD and the complement system have continually identified the presence of complement factors and associated inflammatory proteins within HDL molecules (Table 2). This is discussed in Section 2.3.1. Section 2.3.2 will discuss the genetic associations between HDL-C and AMD.

#### 2.3.1. HDL-C and the Complement System

Past studies into the molecular components of drusen have identified complement factors and associated proteins involved in inflammatory mediation [42,43,44], and it has been suggested that the complement system plays a role in AMD pathogenesis [45,46]. Building on this, studies have attempted to investigate the hypothesis that HDL-C, in its interaction with complement factors, could affect AMD incidence and progression. Upon review of current literature, HDL-C can be said to play a regulatory function in AMD pathogenesis, given its association with many inflammatory mediators involved in both inhibitory and activation pathways with respect to the complement system [47,48]. Dyslipidaemia and altered HDL-C levels may upset this homeostatic balance. Studies have highlighted observations suggesting that the protein content of the HDL particle is dynamic [43]. This suggests that the propensity of HDL particles to be pro or anti-inflammatory can change under specific conditions, and both particulate composition and biological activity of circulating serum HDL-C may be important in AMD pathogenesis.

Some proteomic studies have shown that some HDL particles contain essential complement factors, including C1, C2, C3, C4B, C5 and factor B [42,43], contributing to the pro-inflammatory nature of HDL-C. A composition analysis of HDL-C identified proteins that migrate with HDL-C [41]. Among these proteins were C1q subcomponent subunits B and C (which activate the classical pathway) and ficolin-3 (activates lectin pathway). Complement C1s, C2, C5, factor B and plasma protease C1 inhibitor were also found in HDL-C fractions [41]. Watanabe et al. showed that C9 is also significantly associated with pro-inflammatory HDL (*p*-value < 0.05) [43]. Toomey et al. showed that complement factor H (CFH) and lipoproteins exhibit competitive binding in the sub-RPE extracellular matrix [38]. When CFH is low, lipoproteins accumulate and form sub-RPE deposits. This leads to complement activation, which contributes to RPE damage and visual function impairment. Mechanistically, deposits are due to CFH competition for lipoprotein binding sites in Bruch’s membrane [38]. A 2015 case control study by Paun et al. [33] demonstrated a significant association (*p*-value < 1.9 × 10^−9^) between HDL-C levels and complement system activation level (C3d/C3 ratio) in a case control study.

On the other hand, other studies have suggested that HDL-C also plays a role in complement inhibition. HDL-C associated apolipoprotein E binds to CFH, regulating complement activation [36]. Complement regulatory factors such as CFHR4 and CFHR5 have been found in HDL particles [49,50]. Apolipoprotein A-I (ApoA-I) and Apolipoprotein A-II (ApoA-II) found in HDL-C are also associated with clusterin, an inhibitor of complement-mediated cell lysis via binding to factors C5-C9 [51,52]. In 2015, using affinity chromatography and mass spectrometry, Haapasalo et al. [36] found that CFH interacts with serum apolipoprotein E on HDL particles via FH5-7 domains. Binding of CFH to ApoE prevents excessive alternative pathway activation and protects HDL particles in plasma. They elaborated that HDL-C has a role in reducing alternative pathway activation, at least partially due to binding of complement regulators CFH and clusterin to HDL particles [36].

#### 2.3.2. Genetic Associations between HDL-C and AMD

Genetic associations have been established between increased AMD risk and specific alleles of at least 4 genes encoding components of HDL-C metabolism. They include the Adenosine triphosphate-binding cassette transporter A1 (*ABCA1*), cholesteryl ester transfer protein (*CETP*), apolipoprotein E (*APOE*), and lipoprotein lipase C (*LIPC*) genes [17,26,31,37,53,54,55]. Chen W et al. [40] identified a susceptibility locus for AMD near *TIMP3* (*p*-value = 1.1 × 10^−11^), a metalloproteinase involved in degradation of the extracellular matrix. Data also revealed strong association signals with alleles at two loci (*LIPC*, *p*-value = 1.3 × 10^−7^; *CETP*, *p*-value = 7.4 × 10^−7^) that are associated with serum HDL-C levels. They also reported an association with AMD of HDL-C—associated alleles near *LIPC* (*p*-value = 3.0 × 10^−3^) and *ABCA1* (*p*-value = 5.6 × 10^−4^). Burgess et al. [26] reported that variants in the *CETP* gene region associated with increased circulating HDL-C were also associated with AMD risk, and there is, thus, some genetic evidence that inhibiting *CETP* to increase HDL-C levels may increase AMD risk. *CETP* facilitates transfer of esterified cholesterol between photoreceptor membranes and lipoproteins [56], supporting the high synthesis and degradation of the lipid-rich photoreceptor discs. Lipid balance is maintained by transport of lipoproteins back to Bruch’s membrane [57]. In an ageing retina, the large amount of esterified cholesterol could act as a barrier for lipid transport, thereby facilitating the formation of deposits [58]. Similarly, Cheng et al [37] found a different variant in a known *CETP* lipid gene and novel AMD lipid/cholesterol genes associated with AMD risk in East Asians. Moreover, the *CETP* risk variant was found to interact with high serum HDL levels in individuals of Japanese ancestry and Chinese from Singapore. Ristau et al [39] found variants in *ARMS2, CFH, C3* and *CETP* which were associated with a significantly higher risk for AMD. A common practical problem faced by genetic studies arose in the analysis of rare variants, because the sample size requirements increase with the decrease of allele frequency, leading to difficulties in undertaking a large cohort analysis required for a comprehensive understanding of rare genetic variants and their associations with AMD.

## 3. HDL-C in Age-Related Cataracts

### 3.1. Background of Cataract

Cataract is the loss of optical clarity of the lens, and can be classified based on the location and clinical characteristics of the lens opacities: nuclear, cortical, nuclear and posterior subcapsular [59]. Changes are typically bilateral, but asymmetrical. The elderly population has a greater disposition to cataract development. This has led to the coining of the term “age-related cataracts”. Other links to cataract development have been established with diabetes, hyperglycaemia and myopia [60,61,62,63].

Age-related cataract is a leading cause of vision loss worldwide, placing a significant burden on global healthcare systems. Given the ageing global population, this burden is set to increase substantially [13]. The cost of untreated cataracts, including those stemming from resultant vision loss and social support required, has been estimated to be much greater than the cost of surgical intervention. Hence, regular screening and cataract surgery programmes are deemed to be one of the most cost-effective public-health measures that health authorities can implement.

### 3.2. Epidemiological Evidence

Large population-based observational studies, including the Blue Mountains Eye Study [64] and the Singapore Malay Eye Study (SiMES) [61], among others [59,65,66,67,68,69,70,71,72,73], have attempted to draw epidemiological links between age-related cataract and a range of clinical, environmental and genetic factors. Upon review of current literature, it appears that most studies do report low serum HDL-C concentration as a risk factor for age-related cataract (Table 3). Meyer et al. noted a very strong association between serum HDL-C and the development of lens opacities within the South African population [67]. Subjects with serum HDL-C < 1.5 mmol/L have seven-fold odds of falling in the cataract subgroup compared to those with HDL-C levels ≥ 1.5 mmol/l (OR = 7.33; 95% CI = 2.06–26.10; *p*-value = 0.001) [67]. In 2014, a cross-sectional study of a representative Korean population reported that reduced HDL-C levels were significantly associated with cataract in women (OR = 1.27; 95% CI = 1.07–1.50) [70]. The Blue Mountains Eye Study described that low HDL-C was significantly associated with increased incidence of cortical cataract at 10-year follow-up (HR = 1.57; 95%CI = 1.10–2.24, *p*-value = 0.013) of Australian participants [64]. However, there are recent studies that report insignificant associations between serum HDL-C levels and age-related cataract. In 2018, a cross-sectional study conducted in a Chinese population found that HDL-C levels did not significantly differ between the age-related cataract and normal groups [73]. As part of the Singapore Malay Eye Study, Sabanayagam et al. found a non-significant association between serum HDL-C and cataract [61]. Despite the statistical insignificance of these reports, we note that the direction of association is similar to the aforementioned studies which describe significant results. Pathophysiological mechanisms linking serum HDL-C, or even hyperlipidaemia, with age-related cataract remain unclear, although animal studies have shown that the resultant oxidative stress and inflammation from low HDL-C levels could induce cataract formation [74,75].

## 4. HDL-C in Glaucoma

### 4.1. Background of Glaucoma

Glaucoma is the second most common cause of blindness worldwide [76], characterized by progressive loss of retinal ganglion cells, leading to visual field defects. A timely reduction in intraocular pressure is the primary approach to prevent further glaucomatous damage [77]. There are three major subtypes of glaucoma, including primary open angle glaucoma (POAG), primary angle-closure glaucoma (PACG) and pseudo-exfoliation glaucoma (PEG).

POAG is defined by an open, normal appearing anterior chamber angle, with no other underlying pathologies; while PACG is characterised by a narrow anterior chamber angle. On the other hand, PEG is the most common identifiable secondary glaucoma worldwide [78]. It is the ocular manifestation of pseudo-exfoliation syndrome - a systemic disorder involving deposition of fibrous, extracellular matrix material within the eye.

### 4.2. Epidemiological Evidence

Current literature presents little evidence for a significant, consistent relationship between serum HDL-C levels and glaucoma. Epidemiological links between HDL-C and Glaucoma are summarised in Table 4. In a cross-sectional study, Kurtul et al. reported that serum HDL-C levels did not differ significantly between groups of patients with and without PEG (Control: 1.22 ± 0.39 mmol/L; Glaucoma 1.14 ± 0.28 mmol/L; *p*-value = 0.42) [79]. A population-based study encompassing 16,939 participants from the Korea National Health and Nutrition Examination Survey also observed no significant difference in plasma HDL-C levels between the groups being treated and not being treated for glaucoma [80]. A population-based cross-sectional study involving 3251 Chinese participants reported that dyslipidaemia was not associated with glaucoma [81]. Another cross-sectional study in 2014 described no significant difference in intraocular pressure (IOP) between the group with low HDL-C and the control group (*p* = 0.594) [82]. Perhaps more importantly, a 2018 meta-analysis by Wang et al. reported that low serum HDL-C showed no significant relationship with IOP (Pooled Z = −0.03; 95%CI = −0.06 to 0.01, *p*-value = 0.145, I^2^ = 91.5%) [83].

For papers reporting statistically significant results, most showed an inverse relationship between serum HDL-C levels and IOP. A retrospective, population-based study by Kim et al. involving 80 glaucoma patients and 4015 controls did show that low HDL-C was significantly associated with POAG with normal baseline IOP (OR = 0.96, 95% CI, 0.94–0.99; *p* = 0.004) [84]. A recent cross-sectional study revealed that higher IOP was significantly associated with lower HDL-C (mean IOP difference between low and high HDL-C group = −0.68; 95% CI, −0.99 to −0.36; *p* <0.001) [85]. However, on the other hand, a cross-sectional study in a Japanese population reported that an increase in serum HDL-C by 1 mmol/L was significantly associated with a +0.42 mm Hg IOP change (95% CI = 0.35–0.49, *p* < 0.0001) [86]. Another retrospective study showed a positive correlation between serum HDL-C levels and IOP elevation (Coefficient β (SE) = 0.002 (0.001), *p*-value = 0.001) [87]. Fair comparison of these studies with one another is challenging because of differences in study design, the confounding factors included in the analysis, and the population characteristics among studies.

### 4.3. Laboratory Evidence

Oxidative stress is a phenomenon resulting from an imbalance between reactive oxygen species (ROS) production and defence by antioxidants. Oxidative stress-induced apoptosis of retinal ganglion cells has been implicated in glaucoma pathogenesis [88], while in glaucomatous patients, extensive DNA damage due to oxidative stress has led to trabecular meshwork (TM) injury [89]. Long-term imbalance in aqueous humour composition can also lead to the apoptosis of TM cells and alteration of the optic nerve head [90]; hence, the association between oxidative stress and glaucomatous optic neuropathy [91]. In vivo studies by Sacca et al. reported that both IOP elevation and visual field defects are significantly correlated with the extent of oxidative DNA damage [92]. Given this link between oxidative stress and glaucoma, HDL has been a factor of interest in research due to its various antioxidant effects throughout the body. Circulating HDL particles, primarily HDL-3, protect low-density lipoprotein (LDL) from oxidative damage by free radicals, inhibiting production of pro-inflammatory oxidized lipids. Furthermore, HDL-C also mediates inactivation of lipid hydroperoxides. Lipid hydroperoxides catalyse the oxidative degradation of lipids, which proceeds by a free radical chain reaction mechanism [93]. Studies have been exploring the role of human serum paraoxonase 1 (PON1) in glaucoma. PON1 is a HDL-linked A-esterase glycoenzyme that increases the antioxidant and anti-atherosclerotic potential of HDL-C [94], and is capable of hydrolysing oxidized HDL, LDL and other lipids and esters [95]. Significantly, low PON1 activity is associated with dysfunctional HDL-C [96]. Regarding enzymatic activity towards various substrates, PON1 gene polymorphisms have been shown to affect the performance of PON1 [97,98]. The Q alloenzyme is less efficient in hydrolysing paraoxon, but more efficient toward oxidized lipoproteins than the R alloenzyme [99]. Current literature has stated that although more research is needed to elucidate the role of PON1 function regarding glaucoma, it is reasonable to suppose that PON1 function is clinically important, despite being difficult to prove statistically [91].

### 4.4. Clinical Implications

IOP reduction is currently the only evidence-based treatment for glaucoma. Recent studies have shown that use of statins and other lipid-lowering agents may protect against glaucoma development. Statins inhibit HMGCoA reductase, reducing hepatic cholesterol synthesis and increasing the expression of cell surface LDL receptors. Stein et al. [100] found that those who were prescribed statins experienced a decreased risk of developing POAG from no prior POAG diagnosis, and a decreased risk of converting from a diagnosis of glaucoma suspect to one of POAG. De Castro et al showed that statin use slowed glaucomatous changes to the optic nerve and nerve fibre layer [101]. McGwin et al found that men prescribed statins for ≥ 24 months had 40% reduced odds of developing POAG [102]. In addition, Marcus et al reported that long-term use of statins appeared to be associated with a reduced risk of POAG. However, they hypothesised that this was more likely to be associated with the neuroprotective properties of statins, because the observed effect was independent of the IOP [103]. The effects of statins on *ABCA1* expression have also been explored [104,105], and genome-wide association studies have found variants located upstream of the *ABCA1* gene to be associated with POAG and IOP in multiple populations [106,107,108]. ABCA1 is a transport protein complex. It acts as a cholesterol efflux pump, facilitating cellular lipid removal in reverse cholesterol transport. On chromosome 9q31.1, 4 single-nucleotide polymorphisms (SNPs) (rs2487032 (A), rs2164560 (C), rs2472459 (T) and rs2472519(G) were found to significantly decrease the risk of high-pressure glaucoma (OR = 0.71, 0.73, 0.71, 0.70 respectively) [106]. Mutations in *ABCA1* gene have been associated with Tangier’s disease and familial high-density lipoprotein deficiency [106,107,108]. *ABCA1* is expressed in the TM, iris, ciliary body, cornea, optic nerve, retina and ganglion cell layer [106,107], and could potentially play a role in POAG pathogenesis.

Stein et al. [100] provided a description of proposed mechanisms of how statins could reduce the risk of developing POAG, such as by improving retinal and choroidal perfusion, moderating molecular intermediaries in the aqueous outflow pathways, and exerting neuroprotective effects. However, none of these pathways crucially involve HDL-C as a key component. Perhaps, treatment for dyslipidaemia may be effective against glaucoma. However, upon evaluation of current literature, it is overall unlikely that serum HDL-C plays a major role in glaucoma.

## 5. HDL-C in Diabetic Retinopathy

### 5.1. Background of Diabetic Retinopathy

Diabetic Retinopathy (DR) is a major microangiopathic complication of diabetes, with World Health Organisation estimates placing DR as the cause for approximately 4.8% of cases of vision loss worldwide [109]. Epidemiological studies across various geographical regions have also established DR as the leading cause of visual impairment amongst the middle-aged and, therefore, economically active population [110,111,112].

DR is a disease that affects small retinal vessels, arterioles, capillaries and venules. Endothelial damage leading to vascular lesions increases vessel permeability. Subsequent plasma protein and lipid loss contribute to the clinical signs of retinal edema and exudates. There are two main stages of DR—non-proliferative (NPDR) and proliferative (PDR). The main distinguishing feature is the presence of retinal or optic disc neovascularization in PDR; it is absent in NPDR. NPDR is typically further separated into mild, moderate and severe grades. If left untreated, mild NPDR can advance to moderate and severe NPDR, then to PDR. Concurrent diabetic macular edema (DME), characterized by exudation and edema at the macula (the central focal point of the retina), is a complication [113]. In fact, macular edema is the main cause of visual impairment in subjects with type 2 diabetes mellitus [114]. The International Diabetes Federation has estimated that by 2030, the global tally of individuals with vision-threatening DR, which includes severe NPDR, PDR and Diabetic Macular Edema (DME), will reach 56.3 million.

Risk factors affecting DR onset and progression have been examined extensively, with associations drawn between DR and duration of diabetes, hyperglycaemia, hypertension, high HbA1c and dyslipidaemia [110,115,116]. Landmark studies which have contributed to this knowledge base (non-exhaustive) include the Early Treatment Diabetic Retinopathy Study (ETDRS) [117,118], Diabetic Retinopathy Clinical Research Network (DRCR Net) [119], Blue Mountains Eye Study [120] and Singapore Epidemiology of Eye Diseases (SEED) [121]. While strict maintenance of recommended blood glucose and blood pressure levels provide additional benefits in preventing DR, this is not always possible because of the accompanying risks of hypoglycaemia or hypotension [122,123]. Furthermore, optimized glycaemic control does not preclude occurrence of severe visual damage, despite being effective in limiting onset and progression of DR [124,125]. Identification of alternative or supplementary treatment targets are thus useful in slowing the progression and development of DR [126].

### 5.2. The Relationship between HDL-C and DR

The available literature generally presents an inverse relationship between HDL-C levels and DR, albeit with varying statistical significance [126,127,128,129,130,131,132,133,134,135,136,137,138,139] (Table 5). Hence, serum HDL-C could potentially be a modifiable risk factor of DR, although more evidence is needed to support this.

In the DCCT/EDIC (Diabetes Control and Complications Trial/ Epidemiology of Diabetes Interventions and Complications) study in 2004, more severe retinopathy was significantly associated with lower HDL-C levels in Type 1 Diabetics [134]. A 2019 cross-sectional study by Sasso et al. reported a significant increased odds for DR with elevated HDL-C levels (OR = 1.042; 95% CI = 1.012–1.109; *p*-value = 0.004) upon multivariate logistic regression analysis [137]. A multinational case control study involving 24 centres in 13 countries reported that there was no significant association between a quintile increase of serum HDL-C (≈ 0.2 mmol/L) and odds of DR, although a slight decrease in odds of DR (OR = 0.97; 95% CI = 0.90–1.05; *p*-value = 0.08) [126] was observed upon full adjustment for confounders. The Singapore Malay Eye Study (SiMES) reported an insignificant association between low serum HDL-C and DR [132], while Morton et al. reported no association between baseline HDL-C and the risk of DR or any specific type of retinal event [139].

Some studies investigated the relationship between serum HDL-C and DME. A prospective study involving the DCCT/EDIC cohort [129] later reported that serum HDL-C was insignificantly associated with development of clinically significant macular edema (CSME). However, higher total–to–HDL cholesterol ratio was associated with DR (RR = 3.84, *p*-value = 0.03) [129]. A 2015 meta-analysis investigating dyslipidaemia and DME reported that mean serum HDL-C levels were higher in the presence of DME, but this relationship was not significant [138]. Several hypotheses could explain the variation in result significance. Firstly, while multivariate analysis allows adjustment for covariables, residual confounding risk factors associated with both high HDL-C and DR could exist. These unknowingly omitted confounders would erode strength of analysis. Next, a U-shaped association between HDL-C and DR, resembling that observed in macrovascular disease and mortality, has been suggested [137,140,141,142,143]. In addition, Sacks et al. considered the potential for reverse causation [126], in which a direction of cause-and-effect contrary to common presumption is observed. This phenomenon would be experienced by cross-sectional and observational studies as they cannot distinguish between association and causation.

Regarding genetic relations between HDL-C and DR, a 2017 Mendelian randomization analysis of 60 HDL-related SNPs did not identify a statistically significant odds ratio between HDL-C and DR as a whole or in subgroup analyses [144]. This suggests that associations observed in previous studies may overall be noncausal or perhaps partially due to residual confounders.

### 5.3. Implications on Future Studies

Nevertheless, the review of present literature does provide insight into how future studies can be designed for improved analysis. Firstly, larger study populations, meta-analyses and prospective studies would be helpful to further investigate the relationship between DR and HDL-C levels [126,137]. A large multinational sample size provides the sensitivity and statistical power to identify even a relatively weak association between DR and HDL-C. While HDL-C is a plausible biomarker for DR, studies have also utilised the ApoA-1 to ApoB ratio as a surrogate for the HDL-to-LDL ratio [31]. A large ApoA-1 to ApoB ratio therefore suggests a high level of HDL compared with LDL. Studies have proposed that it offers a more significant relationship with DR, along with higher discriminating abilities, compared to traditional measures of cholesterol [144,145]. The basis for this is because ApoA-I, found in HDL-C, is often overexpressed in the retina of diabetic patients [146], whereas ApoB is a structural protein for VLDL, IDL and LDL [147] and may reflect the atherogenic potential of lipid metabolism [148].

## 6. Conclusions and Future Perspectives

The role of HDL-C in various age-related ocular diseases is widespread and varies, with epidemiological and genetic association studies generating interesting hypotheses involving HDL-C in age-related of ocular conditions. Overall, review of present literature suggests that HDL-C could play a relatively larger role in AMD, age-related cataract and DR, while its association with glaucoma is limited, if any. In AMD, links have been established between HDL-C and components of the complement system or genetic susceptibility loci. Studies have tried to explore a link between HDL-C and glaucoma because of the antioxidative effects of HDL-C and the role of oxidative damage in glaucoma pathogenesis, but to no avail.

HDL particles have distinct physiological and biochemical properties that can be altered through genetic variation, medical therapy, and diet and lifestyle changes. In order to further evaluate these potential areas and their impact on age-related ocular disease, more information needs to be gathered through randomized controlled trials, longitudinal studies and animal models. This is because most of the studies available in current literature are epidemiological in nature, and there is paucity in literature regarding the exact roles & pathophysiological mechanisms in these diseases that involve HDL-C. Ideally, more research into correlations between HDL particle number, size and composition, or specific HDL components in relation to ocular disease would be useful. More detailed knowledge on pathogenic mechanisms of ocular diseases will also provide the basis for exploring HDL-C involvement, and perhaps elucidation of new therapeutic strategies. Until then, continuous efforts must be made to diminish the incidence and progression of age-related ocular diseases.

## Figures and Tables

**Table 1:** HDL-C and AMD—Epidemiological Links from Observational Studies (Selected)

Author (Year)	Study Design	Study Population	Results/Findings
Colijn et al. (2019) [31]	Pooled analysis of cross-sectional data	n = 32,483	Serum HDL was associated with increased odds of AMD (OR = 1.21 per 1-mmol/l increase; 95% CI = 1.14–1.29, *p*-value = 1.35 × 10^−9^).
Saunier et al. (2018) [32]	Population Based Cohort	n = 963 Age ≥ 73 years Adults in Bordeaux, France	Incidence of early AMD was associated with high HDL-C levels (HR = 1.2; 95%CI = 1.0–1.4).
Cheung et al (2017) [27]	Case Control study examining the association between lipoprotein profile and neovascular AMD	n = 193 Adults of Chinese Ethnicity	AMD is associated with increased HDL serum concentration. For each SD increase in serum HDL particles, a 26% increase in nAMD risk was observed (*p* = 0.039). In addition, the increase in HDL particles was mainly driven by an excess of medium-sized particles (8.2–8.8 nm) in participants with nAMD.
Wang et al. (2016) [25]	Meta-Analysis	19 studies n = 82,966	Serum HDL increment of 1 mmol/L significantly increased AMD risk by 18% (RR = 1.18; 95% CI = 1.01–1.35; I^2^ = 53.8%; *p*-value = 0.007).
Paun et al. (2015) [33]	Case Control Cohort	1491 cases, 1579 controls Age ≥ 50 years	AMD patients had significantly higher (p-value = 4.4 × 10^−5^) HDL-C levels. Significant positive correlation between HDL-C and complement activation level (C3d/C3 ratio). (*p*-value < 1.9 × 10^−9^).
Cho et al. (2014) [34]	Population Based Cross Sectional	n = 7899 Age ≥ 40 years Adults in South Korea	Upon multivariate analyses, serum HDL was a significant risk factor for the presence of any AMD type. (Unit of increment = 5 mg/dL (0.13 mmol/L); OR = 1.09; 95% CI 1.02–1.18; *p*-value = 0.019). No correlation with Late AMD
Jonasson et al. (2014) [35]	Population Based Cohort	n = 2868 Adults in Reykjavik, Iceland	In multivariate models, incident AMD was significantly associated with HDL-C. (OR = 1.62 per mmol/L; 95% CI 1.19-2.22; p-value < 0.01). Covariates in regression models included age (in years), female sex (yes/no), current smoker (yes/no), former smoker (yes/no), use of cod liver oil (yes/no), hypertension (yes/no), diabetes mellitus (yes/no), body mass index (kg/m2), total cholesterol (mmol/L), HDL cholesterol (mmol/L) and hsCRP (mg/L).
Klein et al. (2010) [30]	Cross-sectional	n = 2810 Age 21–84 years Beaver Dam Eye Study Adults in USA	Odds of early AMD decreased by approximately 10% per 5mg/dL (0.13 mmol/L) increase in HDL level. (OR = 0.91; 95% CI = 0.83–0.998).
Tan et al. (2007) [29]	Population Based Cohort	n = 3654 Age ≥ 49 years Blue Mountain Eye Study Adults in Australia	Increasing HDL-C was inversely related to incident late AMD (RR per SD increase = 0.74; 95% CI = 0.56–0.99). Elevated total/HDL cholesterol ratio predicted late AMD (RR per SD increase = 1.35; 95% CI = 1.07–1.70) and Geographic atrophy (RR per SD = 1.63; 95% CI = 1.18–2.25).
**AMD, age-related macular degeneration; CI, confidence interval; HDL-C, high-density lipoprotein cholesterol; HR, hazard ratio; hsCRP, high sensitivity C-reactive protein; nAMD, neovascular age-related macular degeneration; OR, odds ratio; RR, relative risk; SD, standard deviation.**			

**Table 2:** HDL-C and AMD—Laboratory Evidence Regarding Possible Pathological Mechanisms (Selected Studies)

Author (Year)	Study Design	Results/Findings
Burgess et al (2017) [26]	2-sample Mendelian randomization to assess known lipid gene associations with AMD risk on 33,536 individuals	Reported that HDL-C is a causal risk factor for AMD (OR = 1.22; 95%CI = 1.03–1.44 per 1 SD increase in HDL-C. Variants in the *CETP* gene region associated with increased circulating HDL-C were also associated with AMD risk, although variants in the LIPC gene region that increase circulating HDL-C have an inverse association. Concluded that there is some genetic evidence that inhibiting *CETP* to increase HDL-C levels may increase AMD risk. The mechanism for an HDL-C/AMD association could be through the modulation of *CETP*.
Haapasalo et al (2015) [36]	Experimental Study	Using affinity chromatography and mass spectrometry, it was found that CFH interacts with serum ApoE via FH5-7 domains. Binding of CFH to ApoE on HDL particles prevents excessive alternative pathway activation and protects HDL particles in plasma. HDL has a role in reducing alternative pathway activation, at least partially due to binding of complement regulators CFH and clusterin to HDL particles.
Cheng et al. (2015) [37]	Genome-wide and exome-wide association study on 2119 patients with exudative AMD and 5691 controls	Reported a strong association between CETP Asp442Gly (rs2303790), an East Asian-specific mutation, and increased risk of AMD (OR = 1.70, *p*-value = 5.60 × 10^−22^). The AMD risk allele (442Gly) increases HDL cholesterol levels by 0.17 mmol/L (*p*-value = 5.82 × 10^−21^) in East Asians (n = 7102).
Toomey et al (2015) [38]	Animal Study (mice). In vivo investigation of sub-RPE deposit formation in aged Cfh+/− and Cfh−/− mice	Complement factor H (CFH) is a major susceptibility gene for AMD. Decreased levels of CFH induce sub-retinal pigmented epithelium (sub-RPE) deposit formation, leading to complement activation, which contributes to RPE damage and visual function impairment. Mechanistically, deposits are due to CFH competition for lipoprotein binding sites in Bruch’s membrane.
Ristau et al (2014) [39]	Analysis of genetic AMD risk polymorphisms and systemic complement activation	Variants in *ARMS2* rs10490924, *CFH* rs1061170, *C3* rs2230199, *C3* rs6795735 and *CETP* rs2230199 were found to be associated with significantly higher risk for AMD.
Chen W et al (2010) [40]	Genome-wide association scan for AMD in 2157 cases and 1150 controls	Identified a susceptibility locus for AMD near TIMP3 (*p*-value = 1.1 × 10^−11^), a metalloproteinase involved in degradation of the extracellular matrix. Data revealed strong association signals with alleles at two loci (LIPC, *p*-value = 1.3 × 10^−7^; *CETP*, *p*-value = 7.4 × 10^−7^) that are associated with serum HDL-C levels. Furthermore, observed an association with AMD of HDL-C—associated alleles near LPL (*p*-value = 3.0 × 10^−3^) and *ABCA1* (*p*-value = 5.6 × 10^−4^).
Gordon et al (2010) [41]	Proteomic Analysis of HDL-C fractionated by Gel Filtration Chromatography	Identified 14 new phospholipid associated proteins that migrate with HDL. These include complement C1q subcomponent subunits B and C which function in activation of the classical pathway and ficolin-3, involved in complement activation via the lectin pathway. Complement C1s, C2, C5, factor B and plasma protease C1 inhibitor, were also found in HDL-C fractions.
**AMD, age-related macular degeneration; ApoE, apolipoprotein E; CI, confidence interval; *CETP*, cholesteryl ester transfer protein; CFH, complement factor H; HDL-C, high-density lipoprotein cholesterol; *LIPC*, lipase C; *LPL*, lipoprotein lipase; OR, odds ratio; RPE, retinal pigment epithelium; RR, relative risk; SD, standard deviation; *TIMP3*, tissue inhibitor of metalloproteinases 3.** **Selected studies are the most recent and relevant.**		

**Table 3:** HDL-C and Age-Related Cataracts—Epidemiological Links from Observational Studies (Selected).

Author (Year)	Study Design	Study Population	Results/Findings
Li S et al. (2018) [73]	Cross sectional case control	219 cases 218 controls age ≥ 45 years Adults in China	HDL-C level did not differ significantly between the age-related cataract group and control group (*p*-value = 0.231). OR not calculated.
Park et al. (2015) [71]	Population based cross-sectional	n = 2852 Age ≥ 40 years Adults in South Korea	Low HDL-C was not significantly associated with any type of cataract.
Park et al. (2014) [70]	Population based cross-sectional	n = 11,076 Age ≥ 31 years Adults in South Korea	Reduced HDL-C levels were significantly associated with cataract in women (OR = 1.27; 95% CI = 1.07–1.50). Upon subgroup analysis, reduced HDL-C levels were significantly associated with nuclear cataract in women (OR = 1.25; 95%CI = 1.03–1.52). Such associations were not found in men.
Ghaem Maralani et al. (2013) [64]	Population-based prospective cohort study10-year follow-up	n = 1997 Age > 48 years Adults in Australia	Low HDL-C was significantly associated with increased incidence of cortical cataract at 10-year follow-up (HR = 1.57; 95%CI = 1.10–2.24, *p*-value = 0.013).
Sabanayagam et al. (2011) [61]	Population-based cross-sectional study	n = 2794 Age 40–80 years Singapore Malay Eye Study Malay Adults in Singapore	Insignificant association found between low-serum HDL-C and cataract. (Multivariate OR = 1.11; 95%CI = 0.88–1.40; *p*-value = 0.4) Low HDL defined as (< 1.0 and < 1.3 mmol/L in male and female, respectively).
Paunksnis et al. (2007) [69]	Population based cross-sectional	n = 1282 Age 35–64 years Adults in Lithuania	Insignificant increase in odds of cataract among women with decreased serum HDL-C (OR = 1.24, 95%CI = 0.77-1.99, *p*-value = 0.426).
Hiller et al. (2003) [68]	Case-control	n = 1684 Age ≥ 45 years Adults in USA	HDL-C < 35 mg/dl (< 0.9051 mmol/L) was significantly associated with decreased risk of Posterior subcapsular cataract in men (OR = 0.97; 95% CI = 0.94-0.99; *p*-value = 0.04). No significant associations noted between serum HDL-C and cortical or nuclear opacities.
Meyer et al. (2003) [67]	Cross-sectional	n = 115 Adults in South Africa	Subjects with serum HDL-C < 1.5 have seven-fold odds of falling in the cataract subgroup compared to those with HDL-C levels ≥ 1.5 mmol/l (OR = 7.33; 95% CI = 2.06–26.10; *p*-value = 0.001). Odds of falling into the cataract subgroup if the individual’s LDL:HDL ratio exceeded 5 was also significantly higher. (OR = 2.35; 95% CI = 1.09–5.04; *p*-value = 0.014).
**CI, confidence interval; HDL-C, high-density lipoprotein cholesterol; HR, hazard ratio; LDL, low-density lipoprotein; OR, odds ratio; RR, relative risk; SD, standard deviation.** **Selected studies are the most recent and relevant.**			

**Table 4:** HDL-C and Glaucoma—Epidemiological Links from Observational Studies (Selected)

Author (Year)	Study Design	Study Population	Results/Findings
Cui et al. (2019) [85]	Population-based cross-sectional	n = 2112 Age ≥ 40 years Adults in Southern China	Multiple regression analysis revealed that higher IOP was significantly associated with lower HDL-C. (Mean IOP difference between low and high HDL-C group = −0.678; 95%CI = −0.993 to −0.363; *p*-value = < 0.001).
Shon et al. (2019) [80]	Population-based cross-sectional	n = 16,939 Age ≥ 40 years Adults in South Korea	No significant difference in plasma HDL-C levels between the groups being treated and not being treated for glaucoma.
Wang et al. (2018) [83]	Meta-analysis	10 studies	Low serum HDL-C showed no significant relationship with IOP. (Pooled Z = −0.03; 95%CI = −0.06 to 0.01, *p*-value = 0.145, I2 = 91.5%).
Kurtul et al. (2017) [79]	Cross-sectional	n = 119	Serum HDL-C levels did not differ significantly between groups of patients with and without pseudo-exfoliation glaucoma (control: 1.22 ± 0.39 mmol/L; glaucoma 1.14 ± 0.28 mmol/L; *p*-value = 0.42).
Yokomichi et al. (2016) [86]	Cross-sectional and longitudinal arms	n = 20,007 (cross-sectional) n = 15,747 (longitudinal) Adults in Japan	Variable of HDL-C, +1 mmol/L was significantly associated with a +0.42 mm Hg IOP change (95% CI = 0.35–0.49, *p*-value < 0.0001).
Kim et al. (2015) [87]	Retrospective cross-sectional	n = 155,198 Age ≥ 20 years Adults in South Korea	After multivariate analysis adjusting for age, sex and other variables, HDL-C and IOP are positively correlated. (Coefficient β (SE) = 0.002 (0.001), *p*-value = 0.001).
Kim MJ et al. (2014) [84]	Retrospective population- based case control	n = 17,901 Age 19–39 years Adults in South Korea	Multivariate analysis found that low HDL-C was significantly associated with primary open angle glaucoma with normal baseline IOP (OR = 0.96, 95%CI = 0.94–0.99; *p*-value = 0.004).
Kim YH et al. (2014) [82]	Population based cross-sectional	n = 4875 Age ≥ 20 years Adult Men in South Korea	No significant difference in IOP between the group with low HDL-C and the control group (*p*-value = 0.594). Other association estimates for HDL-C were not reported.
**CI, confidence interval; HDL-C, high-density lipoprotein cholesterol; HR, hazard ratio; IOP, intraocular pressure; OR, odds ratio; RR, relative risk; SD, standard deviation; SE, standard error.** **Selected studies are the most recent and relevant.**			

**Table 5:** HDL-C and Diabetic Retinopathy—Epidemiological Links from Observational studies (Selected)

Author (Year)	Study Design	Study Population	Results/Findings
Sasso et al. (2019) [137]	Cross-sectional	n = 2068 Age > 14 years Individuals in Italy with Type 2 DM	Diabetic retinopathy was independently associated with HDL-C (OR = 1.042; 95% CI = 1.012–1.109; *p*-value = 0.004). Adjusted for age, HbA1c and duration of diabetes as potential independent risk factors for DR.
Das et al. (2015) [138]	Meta-analysis	21 relevant articles	HDL-C levels were insignificantly higher in the presence of DME (mean difference between groups = 0.0582 mmol/L; 95%CI = −3.62 × 10^−3^ to 0.125, *p*-value = 0.07).
Sacks et al. (2014) [126]	Case-control	n = 2535 Age ≥ 40 years 24 sites in 13 countries Adults with Type 2 DM	Odds ratio for retinopathy associated with a quintile increase of serum HDL-C (≈ 0.2 mmol/L) was insignificant (OR = 0.97; 95% CI = 0.90–1.05; *p*-value = 0.08).
Morton et al. (2012) [139]	Population based prospective cohort	n = 11,140 Age ≥ 55 years Adults with Type 2 DM	No association between baseline HDL-C and the risk of diabetic retinopathy or any specific type of retinal event.
Wong et al. (2008) [132]	Population based Cross-sectional	n = 3261 Age 40–80 years Adults with diabetes of Malay ethnicity in Singapore	Insignificant association between low serum HDL-C and retinopathy (OR = 1.18; 95%; CI = 0.62–2.26; *p*-value = 0.61).
Rema et al. (2006) [131]	Population based Cross-sectional	n = 1736 Age ≥ 20 years Adults with Type 2 DM in Chennai, South India	No significant difference in serum HDL-C levels in Type 2 DM patients with retinopathy compared with those without retinopathy.
Lyons et al. (2004) [134]	Population based Cross-sectional	n = 988 Age 13–39 years DCCT/EDIC Study Adults in USA	ETDRS scoring utilized. Measurement of lipoprotein subclass using nuclear magnetic resonance showed that more severe retinopathy was significantly associated with lower HDL-C levels.
Miljanovic et al (2004) [129]	Population-based prospective study	n = 1441 Age 13–39 years DCCT/EDIC Study Adults in USA	Serum HDL-C was not significantly associated with development of CSME. However, higher total–to–HDL cholesterol ratio (RR = 3.84, 95%CI = 1.58–9.36, *p*-value = 0.03) was significantly associated with higher risk of CSME.
**CI, confidence interval; CSME, clinically significant macular edema; DCCT/EDIC, Diabetes Control and Complications Trial/Epidemiology of Diabetes Interventions and Complications; DM, Diabetes Mellitus; DME, diabetic macular edema; ETDRS, Early Treatment Diabetic Retinopathy Study; HDL-C, high-density lipoprotein cholesterol; HR, hazard ratio; OR, odds ratio; RR, relative risk; SD, standard deviation.** **Selected studies are the most recent and relevant.**			

## References

[1] Asztalos B.F., Schaefer E.J. (2003). High-density lipoprotein subpopulations in pathologic conditions. Am. J. Cardiol..

[2] Connelly M.A., Shalaurova I., Otvos J.D. (2016). High-density lipoprotein and inflammation in cardiovascular disease. Transl. Res..

[3] Kontush A., Lindahl M., Lhomme M., Calabresi L., Chapman M.J., Davidson W.S. (2014). Structure of HDL: Particle Subclasses and Molecular Components. Handb. Exp. Pharmacol..

[4] Rosenson R.S., Brewer H.B., Ansell B., Barter P.J., Chapman M.J., Heinecke J.W., Kontush A., Tall A.R., Webb N.R. (2013). Translation of High-Density Lipoprotein Function Into Clinical Practice. Circulation.

[5] Rosenson R.S., Brewer H.B., Ansell B.J., Barter P., Chapman M.J., Heinecke J.W., Kontush A., Tall A.R., Webb N.R. (2015). Dysfunctional HDL and atherosclerotic cardiovascular disease. Nat. Rev. Cardiol..

[6] Rosenson R.S., Brewer H.B., Chapman M.J., Fazio S., Hussain M.M., Kontush A., Krauss R.M., Otvos J.D., Remaley A.T., Schaefer E.J. (2011). HDL Measures, Particle Heterogeneity, Proposed Nomenclature, and Relation to Atherosclerotic Cardiovascular Events. Clin. Chem..

[7] Rye K.-A., Barter P.J. (2014). Regulation of High-Density Lipoprotein Metabolism. Circ. Res..

[8] Cugati S., Kifley A., Mitchell P., Wang J.J. (2006). Temporal trends in the age-specific prevalence of diabetes and diabetic retinopathy in older persons: Population-based survey findings. Diabetes Res. Clin. Pract..

[9] Tham Y.C., Li X., Wong T.Y., Quigley H.A., Aung T., Cheng C.-Y. (2014). Global Prevalence of Glaucoma and Projections of Glaucoma Burden through 2040. Ophthalmology.

[10] Wong W.L., Su X., Li X., Cheung C.M.G., Klein A.P., Cheng C.-Y., Wong T.Y. (2014). Global prevalence of age-related macular degeneration and disease burden projection for 2020 and 2040: A systematic review and meta-analysis. Lancet Glob. Heal..

[11] Flaxman S.R., Bourne R.R.A., Resnikoff S., Ackland P., Braithwaite T., Cicinelli M.V., Das A., Jonas J.B., Keeffe J., Kempen J.H. (2017). Global causes of blindness and distance vision impairment 1990–2020: A systematic review and meta-analysis. Lancet Glob. Health.

[12] Lim L.S., Mitchell P., Seddon J.M., Holz F.G., Wong T.Y. (2012). Age-related macular degeneration. Lancet.

[13] A Bourne R.R., Stevens G.A., A White R., Smith J.L., Flaxman S.R., Price H., Jonas J.B., Keeffe J., Leasher J., Naidoo K. (2013). Causes of vision loss worldwide, 1990–2010: A systematic analysis. Lancet Glob. Heal..

[14] Lee P., Feldman Z.W., Ostermann J., Brown D.S., Sloan F. (2003). Longitudinal Prevalence of Major Eye Diseases. Arch. Ophthalmol..

[15] Jager R.D., Mieler W.F., Miller J.W. (2008). Age-Related Macular Degeneration. N. Engl. J. Med..

[16] DeAngelis M.M., Owen L.A., Morrison M.A., Morgan D.J., Li M., Shakoor A., Vitale A., Iyengar S., Stambolian D., Kim I.K. (2017). Genetics of age-related macular degeneration (AMD). Hum. Mol. Genet..

[17] Fritsche L.G., Fariss R.N., Stambolian D., Abecasis G., Curcio C.A., Swaroop A. (2014). Age-related macular degeneration: Genetics and biology coming together. Annu. Rev. Genom. Hum. Genet..

[18] A Curcio C., Millican C.L. (1999). Basal linear deposit and large drusen are specific for early age-related maculopathy. Arch. Ophthalmol..

[19] Green W.R., Enger C. (2005). Age-related macular degeneration histopathologic studies: The 1992 Lorenz E. Zimmerman Lecture. 1992. Retina.

[20] Sarks J.P., Sarks S.H., Killingsworth M.C. (1988). Evolution of geographic atrophy of the retinal pigment epithelium. Eye.

[21] Balaratnasingam C., Yannuzzi L.A., Curcio C.A., Morgan W.H., Querques G., Capuano V., Souied E., Jung J., Freund K.B. (2016). Associations Between Retinal Pigment Epithelium and Drusen Volume Changes During the Lifecycle of Large Drusenoid Pigment Epithelial Detachments. Investig. Opthalmol. Vis. Sci..

[22] Ferris F.L., Wilkinson C., Bird A., Chakravarthy U., Chew E., Csaky K.G., Sadda S.R. (2013). Clinical Classification of Age-related Macular Degeneration. Ophthalmology.

[23] Klein A.P., Sharrett A.R., Klein B.E., Moss S.E., Folsom A.R., Wong T.Y., Brancati F.L., Hubbard L.D., Couper D. (2002). The association of atherosclerosis, vascular risk factors, and retinopathy in adults with diabetes: The atherosclerosis risk in communities study. Ophthalmology.

[24] Kersten E., Paun C.C., Schellevis R., Hoyng C.B., Delcourt C., Lengyel I., Peto T., Ueffing M., Klaver C.C.W., Dammeier S. (2018). Systemic and ocular fluid compounds as potential biomarkers in age-related macular degeneration. Surv. Ophthalmol..

[25] Wang Y., Wang M., Zhang X., Zhang Q., Nie J., Zhang M., Liu X., Ma L. (2016). The Association between the Lipids Levels in Blood and Risk of Age-Related Macular Degeneration. Nutrients.

[26] Burgess S., Smith G.D. (2017). Mendelian Randomization Implicates High-Density Lipoprotein Cholesterol-Associated Mechanisms in Etiology of Age-Related Macular Degeneration. Ophthalmology.

[27] Cheung C.M.G., Gan A., Fan Q., Chee M.L., Apte R.S., Khor C.C., Yeo I., Mathur R., Cheng C.-Y., Wong T.Y. (2017). Plasma lipoprotein subfraction concentrations are associated with lipid metabolism and age-related macular degeneration. J. Lipid Res..

[28] Cougnard-Grégoire A., Delyfer M.-N., Korobelnik J.-F., Rougier M.-B., Le Goff M., Dartigues J.-F., Barberger-Gateau P., Delcourt C. (2014). Elevated High-Density Lipoprotein Cholesterol and Age-Related Macular Degeneration: The Alienor Study. PLoS ONE.

[29] Tan J.S., Mitchell P., Smith W., Wang J.J. (2007). Cardiovascular Risk Factors and the Long-term Incidence of Age-Related Macular Degeneration. Ophthalmology.

[30] Klein R., Cruickshanks K.J., Nash S.D., Krantz E.M., Nieto F.J., Huang G.H., Pankow J.S., Klein B.E.K. (2010). The Prevalence of Age-Related Macular Degeneration and Associated Risk Factors. Arch. Ophthalmol..

[31] Colijn J.M., Hollander A.I.D., Demirkan A., Cougnard-Grégoire A., Verzijden T., Kersten E., Meester-Smoor M.A., Merle B.M., Papageorgiou G., Ahmad S. (2019). Increased High-Density Lipoprotein Levels Associated with Age-Related Macular Degeneration. Ophthalmology.

[32] Saunier V., Merle B.M., Delyfer M.-N., Cougnard-Grégoire A., Rougier M.-B., Amouyel P., Lambert J.-C., Dartigues J.-F., Korobelnik J.-F., Delcourt C. (2018). Incidence of and Risk Factors Associated with Age-Related Macular Degeneration. JAMA Ophthalmol..

[33] Paun C.C., Ersoy L., Schick T., Groenewoud J.M.M., Lechanteur Y.T., Fauser S., Hoyng C., De Jong E.K., Hollander A.I.D. (2015). Genetic Variants and Systemic Complement Activation Levels Are Associated With Serum Lipoprotein Levels in Age-Related Macular Degeneration. Investig. Opthalmol. Vis. Sci..

[34] Cho B.-J., Heo J., Kim T.W., Ahn J., Chung H. (2014). Prevalence and Risk Factors of Age-Related Macular Degeneration in Korea: The Korea National Health and Nutrition Examination Survey 2010–2011. Investig. Opthalmol. Vis. Sci..

[35] Jonasson F., Fisher D.E., Eiriksdottir G., Sigurdsson S., Klein R., Launer L.J., Harris T., Gudnason V., Cotch M.F. (2014). Five-year incidence, progression, and risk factors for age-related macular degeneration: The age, gene/environment susceptibility study. Ophthalmology.

[36] Haapasalo K., Van Kessel K., Nissila E., Metso J., Johansson T., Miettinen S., Varjosalo M., Kirveskari J., Kuusela P., Chroni A. (2015). Complement Factor H Binds to Human Serum Apolipoprotein E and Mediates Complement Regulation on High Density Lipoprotein Particles. J. Boil. Chem..

[37] Cheng C.-Y., Yamashiro K., Chen L.J., Ahn J., Huang L., Huang L., Cheung C.M.G., Miyake M., Cackett P.D., Yeo I.Y. (2015). New loci and coding variants confer risk for age-related macular degeneration in East Asians. Nat. Commun..

[38] Toomey C.B., Kelly U., Saban D.R., Rickman C.B. (2015). Regulation of age-related macular degeneration-like pathology by complement factor H. Proc. Natl. Acad. Sci. USA.

[39] Ristau T., Paun C., Ersoy L., Hahn M., Lechanteur Y., Hoyng C., de Jong E.K., Daha M.R., Kirchhof B., den Hollander A.I. (2014). Impact of the common genetic associations of age-related macular degeneration upon systemic complement Component C3d Levels. PLoS ONE.

[40] Chen W., Stambolian D., Edwards A.O., Branham K.E., Othman M., Jakobsdottir J., Tosakulwong N., Pericak-Vance M.A., Campochiaro P.A., Klein M.L. (2010). Genetic variants near TIMP3 and high-density lipoprotein-associated loci influence susceptibility to age-related macular degeneration. Proc. Natl. Acad. Sci. USA.

[41] Gordon S.M., Deng J., Lu L.J., Davidson W.S. (2010). Proteomic Characterization of Human Plasma High Density Lipoprotein Fractionated by Gel Filtration Chromatography. J. Proteome Res..

[42] Rezaee F., Casetta B., Levels J.H.M., Speijer D., Meijers J.C.M. (2006). Proteomic analysis of high-density lipoprotein. Proteomics.

[43] Watanabe J., Charles-Schoeman C., Miao Y., Elashoff D., Lee Y.Y., Katselis G., Lee T.D., Reddy S.T. (2012). Proteomic profiling following immunoaffinity capture of high-density lipoprotein: Association of acute-phase proteins and complement factors with proinflammatory high-density lipoprotein in rheumatoid arthritis. Arthritis Rheum..

[44] Johnson L.V., Ozaki S., Staples M.K., A Erickson P., Anderson D.H. (2000). A Potential Role for Immune Complex Pathogenesis in Drusen Formation. Exp. Eye Res..

[45] Johnson L.V., Leitner W.P., Staples M.K., Anderson D.H. (2001). Complement Activation and Inflammatory Processes in Drusen Formation and Age Related Macular Degeneration. Exp. Eye Res..

[46] Hageman G.S. (2001). An Integrated Hypothesis That Considers Drusen as Biomarkers of Immune-Mediated Processes at the RPE-Bruch’s Membrane Interface in Aging and Age-Related Macular Degeneration. Prog. Retin. Eye Res..

[47] Eren E., Yilmaz N., Aydin O. (2012). High Density Lipoprotein and it’s Dysfunction. Open Biochem. J..

[48] Rao V.S., Kakkar V.V. (2010). Friend Turns Foe: Transformation of Anti-Inflammatory HDL to Proinflammatory HDL during Acute-Phase Response. Cholesterol.

[49] Skerka C., Hellwage J., Weber W., Tilkorn A., Buck F., Marti T., Kampen E., Beisiegel U., Zipfel P.F. (1997). The human factor H-related protein 4 (FHR-4). A novel short consensus repeat-containing protein is associated with human triglyceride-rich lipoproteins. J. Boil. Chem..

[50] McRae J.L., Duthy T.G., Griggs K.M., Ormsby R.J., Cowan P.J., Cromer B.A., McKinstry W., Parker M.W., Murphy B.F., Gordon D.L. (2005). Human factor H-related protein 5 has cofactor activity, inhibits C3 convertase activity, binds heparin and C-reactive protein, and associates with lipoprotein. J. Immunol..

[51] Rosenfeld S.I., Packman C.H., Leddy J.P. (1983). Inhibition of the Lytic Action of Cell-bound Terminal Complement Components by Human High Density Lipoproteins and Apoproteins. J. Clin. Investig..

[52] Tschopp J., Chonn A., Hertig S., French L. (1993). Clusterin, the human apolipoprotein and complement inhibitor, binds to complement C7, C8 beta, and the b domain of C9. J. Immunol..

[53] Wang D., Zhou J., Hou X., Nguyen D.H., Cao G., Li G., Qiu G., Zhang K., Zhang M., Su Z. (2014). CETP Gene may be Associated with Advanced Age-Related Macular Degeneration in the Chinese Population. Ophthalmic Genet..

[54] Wang Y.-F., Han Y., Zhang R., Qin L., Wang M.-X., Ma L. (2015). CETP/LPL/LIPC gene polymorphisms and susceptibility to age-related macular degeneration. Sci. Rep..

[55] Neale B.M., Fagerness J., Reynolds R., Sobrin L., Parker M., Raychaudhuri S., Tan P.L., Oh E.C., Merriam J.E., Souied E. (2010). Genome-wide association study of advanced age-related macular degeneration identifies a role of the hepatic lipase gene (LIPC). Proc. Natl. Acad. Sci. USA.

[56] Tserentsoodol N., Gordiyenko N.V., Pascual I., Lee J.W., Fliesler S.J., Rodriguez I. (2006). Intraretinal lipid transport is dependent on high density lipoprotein-like particles and class B scavenger receptors. Mol. Vis..

[57] Johnson L.V., Forest D.L., Banna C.D., Radeke C.M., Maloney M.A., Hu J., Spencer C.N., Walker A.M., Tsie M.S., Bok D. (2011). Cell culture model that mimics drusen formation and triggers complement activation associated with age-related macular degeneration. Proc. Natl. Acad. Sci. USA.

[58] Curcio C.A., Johnson M., Rudolf M., Huang J.-D. (2011). The oil spill in ageing Bruch membrane. Br. J. Ophthalmol..

[59] Klein B.E., Klein A.P., Linton K.L. (1992). Prevalence of Age-related Lens Opacities in a Population. Ophthalmology.

[60] Tang Y., Wang X., Wang J., Huang W., Gao Y., Luo Y., Yang J., Lu Y. (2016). Prevalence of Age-Related Cataract and Cataract Surgery in a Chinese Adult Population: The Taizhou Eye Study. Investig. Opthalmol. Vis. Sci..

[61] Sabanayagam C., Wang J.J., Mitchell P., Tan A.G., Tai E.S., Aung T., Saw S.M., Wong T.Y. (2011). Metabolic syndrome components and age-related cataract: The Singapore Malay eye study. Investig. Ophthalmol. Vis. Sci..

[62] Jacques P.F., Moeller S.M., E Hankinson S., Chylack L.T., Rogers G., Tung W., Wolfe J.K., Willett W.C., Taylor A. (2003). Weight status, abdominal adiposity, diabetes, and early age-related lens opacities. Am. J. Clin. Nutr..

[63] Hennis A., Wu S.-Y., Nemesure B., Leske M.C. (2004). Risk Factors for Incident Cortical and Posterior Subcapsular Lens Opacitiesin the Barbados Eye Studies. Arch. Ophthalmol..

[64] Maralani H.G., Tai B.C., Wong T.Y., Tai E.S., Li J., Wang J.J., Mitchell P. (2013). Metabolic Syndrome and Risk of Age-Related Cataract over Time: An Analysis of Interval-Censored Data Using a Random-Effects Model. Investig. Opthalmol. Vis. Sci..

[65] Klein B.E., Klein A.P., Lee K.E. (1997). Cardiovascular Disease, Selected Cardiovascular Disease Risk Factors, and Age-related Cataracts: The Beaver Dam Eye Study. Am. J. Ophthalmol..

[66] Drinkwater J.J., Davis T.M.E., Turner A.W., Bruce D.G., Davis W.A. (2018). Incidence and Determinants of Intraocular Lens Implantation in Type 2 Diabetes: The Fremantle Diabetes Study Phase II. Diabetes Care.

[67] Meyer D., Parkin D., Maritz F.J., Liebenberg P.H. (2003). Abnormal serum lipoprotein levels as a risk factor for the development of human lenticular opacities. Cardiovasc. J. South Afr. Off. J. South. Afr. Card. Soc. South Afr. Soc. Card. Prac..

[68] Hiller R., Sperduto R.D., Reed G.F., D’Agostino R.B., Wilson P.W.F. (2003). Serum lipids and age-related lens opacities: A longitudinal investigation. Ophthalmology.

[69] Paunksnis A., Bojarskiene F., Cimbalas A., Cerniauskiene L., Luksiene D., Tamosiunas A. (2007). Relation between cataract and metabolic syndrome and its components. Eur. J. Ophthalmol..

[70] Park Y.-H., Shin J.A., Han K., Yim H.W., Lee W.-C., Park Y.-M. (2014). Gender Difference in the Association of Metabolic Syndrome and Its Components with Age-Related Cataract: The Korea National Health and Nutrition Examination Survey 2008–2010. PLoS ONE.

[71] Park S., Lee E.-H. (2015). Association between metabolic syndrome and age-related cataract. Int. J. Ophthalmol..

[72] A Kahn H., Leibowitz H.M., Ganley J.P., Kini M.M., Colton T., Nickerson R.S., Dawber T.R. (1977). The Framingham Eye Study. I. Outline and major prevalence findings. Am. J. Epidemiol..

[73] Li S., Li D., Zhang Y., Teng J., Shao M., Cao W. (2018). Association between serum lipids concentration and patients with age-related cataract in China: A cross-sectional, case–control study. BMJ Open.

[74] Von Eckardstein A., Hersberger M., Rohrer L. (2005). Current understanding of the metabolism and biological actions of HDL. Curr. Opin. Clin. Nutr. Metab. Care.

[75] Tsutsumi K., Inoue Y., Yoshida C. (1999). Acceleration of Development of Diabetic Cataract by Hyperlipidemia and Low High-Density Lipoprotein in Rats. Boil. Pharm. Bull..

[76] Quigley H.A., Broman A.T. (2006). The number of people with glaucoma worldwide in 2010 and 2020. Br. J. Ophthalmol..

[77] E Pillunat L., Erb C., Jünemann A.G., Kimmich F. (2017). Micro-invasive glaucoma surgery (MIGS): A review of surgical procedures using stents. Clin. Ophthalmol..

[78] Ritch R. (1994). Exfoliation Syndrome—The Most Common Identifiable Cause of Open-Angle Glaucoma. J. Glaucoma.

[79] Kurtul B.E., Kurtul A., Ozer P.A., Kabataş E.U., Ertugrul G.T. (2015). Serum Lipid Levels in Pseudoexfoliation Syndrome. Semin. Ophthalmol..

[80] Shon K., Sung K.R. (2019). Dyslipidemia, Dyslipidemia Treatment, and Open-angle Glaucoma in the Korean National Health and Nutrition Examination Survey. J. Glaucoma.

[81] Wang S., Xu L., Jonas J.B., Wang Y.X., You Q.S., Yang H. (2012). Dyslipidemia and Eye Diseases in the Adult Chinese Population: The Beijing Eye Study. PLoS ONE.

[82] Kim Y.-H., Jung S.W., Nam G.-E., Han K.D., Bok A.R., Baek S.J., Cho K.-H., Choi Y.S., Kim S.M., Ju S.Y. (2014). High intraocular pressure is associated with cardiometabolic risk factors in South Korean men: Korean National Health and Nutrition Examination Survey, 2008–2010. Eye.

[83] Wang Y.-X., Tao J.-X., Yao Y. (2019). The association of intraocular pressure with metabolic syndrome and its components: A Meta-analysis and systematic review. Int. J. Ophthalmol..

[84] Kim M.J., Kim M.J., Kim H.S., Jeoung J.W., Park K.H. (2014). Risk factors for open-angle glaucoma with normal baseline intraocular pressure in a young population: The Korea National Health and Nutrition Examination Survey. Clin. Exp. Ophthalmol..

[85] Cui Y., Yang X., Zhang G., Guo H., Zhang M., Zhang L., Zeng J., Liu Q., Zhang L., Meng Q. (2019). Intraocular Pressure in General and Diabetic Populations from Southern China: The Dongguan Eye Study. Investig. Opthalmol. Vis. Sci..

[86] Yokomichi H., Kashiwagi K., Kitamura K., Yoda Y., Tsuji M., Mochizuki M., Sato M., Shinohara R., Mizorogi S., Suzuki K. (2016). Evaluation of the associations between changes in intraocular pressure and metabolic syndrome parameters: A retrospective cohort study in Japan. BMJ Open.

[87] Kim Y.J., Chun Y.S., Lee M.Y., Kim J.M., Shim S.H., Yoo C., Bae J.H., Park K.H. (2015). Association of IOP with Systemic Factors in a Korean Cohort. Optom. Vis. Sci..

[88] Wang C., Ren Y.-L., Zhai J., Zhou X.-Y., Wu J. (2019). Down-regulated LAMA4 inhibits oxidative stress-induced apoptosis of retinal ganglion cells through the MAPK signaling pathway in rats with glaucoma. Cell Cycle.

[89] Izzotti A., Bagnis A., Sacca S. (2006). The role of oxidative stress in glaucoma. Mutat. Res..

[90] Ciotu I.M., Stoian I., Gaman L., Popescu M.V., Atanasiu V. (2015). Biochemical changes and treatment in glaucoma. J. Med. Life.

[91] Yilmaz N., Coban D.T., Bayindir A., Erol M.K., Ellidag H.Y., Giray Özlem, Sayrac S., Tekeli S.O., Eren E. (2015). Higher serum lipids and oxidative stress in patients with normal tension glaucoma, but not pseudoexfoliative glaucoma. Bosn. J. Basic Med Sci..

[92] Sacca S., Pascotto A., Camicione P., Capris P., Izzotti A. (2005). Oxidative DNA Damage in the Human Trabecular Meshwork. Arch. Ophthalmol..

[93] Brites F., Martin M., Guillas I., Kontush A. (2017). Antioxidative activity of high-density lipoprotein (HDL): Mechanistic insights into potential clinical benefit. BBA Clin..

[94] Yilmaz N. (2012). Relationship between paraoxonase and homocysteine: Crossroads of oxidative diseases. Arch. Med Sci..

[95] Mackness M., Mackness B. (2015). Human paraoxonase-1 (PON1): Gene structure and expression, promiscuous activities and multiple physiological roles. Gene.

[96] Eren E., Ellidag H.Y., Aydin O., Yilmaz N. (2015). HDL functionality and crystal-based sterile inflammation in atherosclerosis. Clin. Chim. Acta.

[97] Eren E., Yılmaz N., Aydin O., Ellidağ H.Y. (2014). Anticipatory Role of High Density Lipoprotein and Endothelial Dysfunction: An Overview. Open Biochem. J..

[98] Eren E., Ellidag H.Y., Aydin O., Yılmaz N. (2014). Homocysteine, Paraoxonase-1 and Vascular Endothelial Dysfunction: Omnibus viis Romam Pervenitur. J. Clin. Diagn. Res..

[99] M A., Hardak E., Vaya J., Mahmood S., Milo S., Hoffman A., Billicke S., Draganov D., Rosenblat M. (2000). Human serum paraoxonases (PON1) Q and R selectively decrease lipid peroxides in human coronary and carotid atherosclerotic lesions: PON1 esterase and peroxidase-like activities. Circulation.

[100] Stein J.D., Newman-Casey P.A., Talwar N., Nan B., Richards J.E., Musch D.C. (2012). The relationship between statin use and open-angle glaucoma. Ophthalmology.

[101] De Castro D.K., Punjabi O.S., Bostrom A.G., Stamper R.L., Lietman T.M., Ray K., Lin S.C. (2007). Effect of statin drugs and aspirin on progression in open-angle glaucoma suspects using confocal scanning laser ophthalmoscopy. Clin. Exp. Ophthalmol..

[102] McGwin G., McNeal S., Owsley C., Girkin C., Epstein D., Lee P.P. (2004). Statins and other cholesterol-lowering medications and the presence of glaucoma. Arch Ophthalmol..

[103] Marcus M.W., Müskens R.P.H.M., Ramdas W.D., Wolfs R.C.W., De Jong P.T.V.M., Vingerling J.R., Hofman A., Stricker B.H., Jansonius N.M. (2012). Cholesterol-Lowering Drugs and Incident Open-Angle Glaucoma: A Population-Based Cohort Study. PLoS ONE.

[104] Seeree P., Janvilisri T., Kangsamaksin T., Tohtong R., Kumkate S. (2019). Downregulation of ABCA1 and ABCG1 transporters by simvastatin in cholangiocarcinoma cells. Oncol. Lett..

[105] Wong J., Quinn C.M., Gelissen I.C., Jessup W., Brown A.J. (2008). The effect of statins on ABCA1 and ABCG1 expression in human macrophages is influenced by cellular cholesterol levels and extent of differentiation. Atherosclerosis.

[106] Chen Y., Lin Y., Vithana E.N., Jia L., Zuo X., Wong T.Y., Chen L.J., Zhu X., Tam P.O.S., Gong B. (2014). Common variants near ABCA1 and in PMM2 are associated with primary open-angle glaucoma. Nat. Genet..

[107] Gharahkhani P., Burdon K.P., Fogarty R., Sharma S., Hewitt A.W., Martin S., Law M.H., Cremin K., Bailey J.N.C., Wellcome Trust Case Control Consortium 2 (2014). Common variants near ABCA1, AFAP1 and GMDS confer risk of primary open-angle glaucoma. Nat. Genet..

[108] Luo H., Chen Y., Ye Z., Sun X., Shi Y., Luo Q., Gong B., Shuai P., Yang J., Zhou Y. (2015). Evaluation of the Association Between Common Genetic Variants Near theABCA1Gene and Primary Angle Closure Glaucoma in a Han Chinese Population. Investig. Opthalmol. Vis. Sci..

[109] Resnikoff S., Pascolini D., Etya’Ale D., Kocur I., Pararajasegaram R., Pokharel G.P., Mariotti S.P. (2004). Global data on visual impairment in the year 2002. Bull. World Heal. Organ..

[110] Yau J.W., Rogers S.L., Kawasaki R., Lamoureux E.L., Kowalski J.W., Bek T., Chen S.-J., Dekker J.M., Fletcher A., Grauslund J. (2012). Global Prevalence and Major Risk Factors of Diabetic Retinopathy. Diabetes Care.

[111] Bruno G., Bonora E., Miccoli R., Vaccaro O., Rossi E., Bernardi D., De Rosa M., Marchesini G. (2012). Quality of Diabetes Care in Italy: Information From a Large Population-Based Multiregional Observatory (ARNO Diabetes). Diabetes Care.

[112] Semeraro F., Cancarini A., Dell’Omo R., Rezzola S., Romano M.R., Costagliola C. (2015). Diabetic Retinopathy: Vascular and Inflammatory Disease. J. Diabetes Res..

[113] Wong T.Y., Cheung C.M.G., Larsen M., Sharma S., Simó R. (2016). Diabetic retinopathy. Nat. Rev. Dis. Prim..

[114] El Hindy N., Ong J.M. (2010). Asymmetric diabetic retinopathy. J. Diabetes.

[115] Klein A.P. (2007). Overview of Epidemiologic Studies of Diabetic Retinopathy. Ophthalmic Epidemiol..

[116] Stratton I., Kohner E.M., Aldington S., Turner R.C., Holman R.R., Manley S.E., Matthews D.R. (2001). UKPDS 50: Risk factors for incidence and progression of retinopathy in Type II diabetes over 6 years from diagnosis. Diabetologia.

[117] Chew E.Y., Klein M.L., Ferris F.L., A Remaley N., Murphy R.P., Chantry K., Hoogwerf B.J., Miller D. (1996). Association of elevated serum lipid levels with retinal hard exudate in diabetic retinopathy. Early Treatment Diabetic Retinopathy Study (ETDRS) Report 22. Arch. Ophthalmol..

[118] Davis M.D., Fisher M.R., E Gangnon R., Barton F., Aiello L.M., Chew E.Y., Ferris F.L., Knatterud G.L. (1998). Risk factors for high-risk proliferative diabetic retinopathy and severe visual loss: Early Treatment Diabetic Retinopathy Study Report #18. Investig. Ophthalmol. Vis. Sci..

[119] Sun J.K., Jampol L.M. (2019). The Diabetic Retinopathy Clinical Research Network (DRCR.net) and Its Contributions to the Treatment of Diabetic Retinopathy. Ophthalmic Res..

[120] Cikamatana L., Mitchell P., Rochtchina E., Foran S., Wang J.J. (2007). Five-year incidence and progression of diabetic retinopathy in a defined older population: The Blue Mountains Eye Study. Eye.

[121] Tan G.S., Gan A., Charumathi S., Tham Y.C., Neelam K., Mitchell P., Wang J.J., Lamoureux E.L., Cheng C.-Y., Wong T.Y. (2018). Ethnic Differences in the Prevalence and Risk Factors of Diabetic Retinopathy. Ophthalmology.

[122] Chew E.Y., Ambrosius W.T., Davis M.D., Danis R.P., Gangaputra S., Greven C.M., Hubbard L., A Esser B., ACCORD Study Group, ACCORD Eye Study Group (2010). Effects of medical therapies on retinopathy progression in type 2 diabetes. New Engl. J. Med..

[123] Cushman W.C., Evans G.W., Byington R.P., Goff D.C., Grimm R.H., Cutler J.A., Simons-Morton D.G., Basile J.N., Corson M.A., ACCORD Study Group (2010). Effects of intensive blood-pressure control in type 2 diabetes mellitus. New Engl. J. Med..

[124] Boussageon R., Bejan-Angoulvant T., Saadatian-Elahi M., Lafont S., Bergeonneau C., Kassai B., Erpeldinger S., Wright J.M., Gueyffier F., Cornu C. (2011). Effect of intensive glucose lowering treatment on all cause mortality, cardiovascular death, and microvascular events in type 2 diabetes: Meta-analysis of randomised controlled trials. BMJ.

[125] Hemmingsen B., Lund S.S., Gluud C., Vaag A., Almdal T., Hemmingsen C., Wetterslev J. (2011). Intensive glycaemic control for patients with type 2 diabetes: Systematic review with meta-analysis and trial sequential analysis of randomised clinical trials. BMJ.

[126] Sacks F.M., Hermans M.P., Fioretto P., Valensi P., Davis T., Horton E., Wanner C., Al-Rubeaan K., Aronson R., Barzon I. (2013). Association Between Plasma Triglycerides and High-Density Lipoprotein Cholesterol and Microvascular Kidney Disease and Retinopathy in Type 2 Diabetes Mellitus: A Global Case-Control Study in 13 Countries. Circulation.

[127] Benarous R., Sasongko M.B., Qureshi S., Fenwick E., Dirani M., Wong T.Y., Lamoureux E.L. (2011). Differential Association of Serum Lipids with Diabetic Retinopathy and Diabetic Macular Edema. Investig. Opthalmol. Vis. Sci..

[128] Klein B.E., Moss S.E., Klein R., Surawicz T.S. (1991). The Wisconsin Epidemiologic Study of Diabetic Retinopathy. XIII. Relationship of serum cholesterol to retinopathy and hard exudate. Ophthalmology.

[129] Miljanovic B., Glynn R.J., Nathan D.M., Manson J.E., Schaumberg D.A. (2004). A prospective study of serum lipids and risk of diabetic macular edema in type 1 diabetes. Diabetes.

[130] Raman R., Rani P.K., Kulothungan V., Rachepalle S.R., Kumaramanickavel G., Sharma T. (2010). Influence of Serum Lipids on Clinically Significant versus Nonclinically Significant Macular Edema. Ophthalmology.

[131] Rema M., Srivastava B.K., Anitha B., Deepa R., Mohan V. (2006). Association of serum lipids with diabetic retinopathy in urban South Indians—The Chennai Urban Rural Epidemiology Study (CURES) Eye Study—2. Diabet. Med..

[132] Wong T.Y., Cheung N., Tay W.T., Wang J.J., Aung T., Saw S.M., Lim S.C., Tai E.S., Mitchell P. (2008). Prevalence and Risk Factors for Diabetic Retinopathy. Ophthalmology.

[133] Sachdev N., Sahni A. (2010). Association of systemic risk factors with the severity of retinal hard exudates in a north Indian population with type 2 diabetes. J. Postgrad. Med..

[134] Lyons T.J., Jenkins A.J., Zheng D., Lackland D.T., McGee D., Garvey W.T., Klein R.L. (2004). Diabetic retinopathy and serum lipoprotein subclasses in the DCCT/EDIC cohort. Investig. Opthalmol. Vis. Sci..

[135] Cezario S.M., Calastri M.C.J., Oliveira C.I.F., Carmo T.S.D., Pinhel M.A.D.S., De Godoy M.F., Jorge R., Cotrim C.C., Souza D.R.S., Siqueira R.C. (2015). Association of high-density lipoprotein and apolipoprotein E genetic variants with age-related macular degeneration. Arq. Bras. Oftalmol..

[136] Misra A., Kumar S., Vikram N.K., Kumar A. (2003). The role of lipids in the development of diabetic microvascular complications: Implications for therapy. Am. J. Cardiovasc. Drugs.

[137] Sasso F.C., Pafundi P.C., Gelso A., Bono V., Costagliola C., Marfella R., Sardu C., Rinaldi L., Galiero R., Acierno C. (2019). High HDL cholesterol: A risk factor for diabetic retinopathy? Findings from NO BLIND study. Diabetes Res. Clin. Pract..

[138] Das R., Kerr R., Chakravarthy U., Hogg R.E. (2015). Dyslipidemia and Diabetic Macular Edema. Ophthalmology.

[139] Morton J., Zoungas S., Li Q., Patel A.A., Chalmers J., Woodward M., Celermajer D., Beulens J.W., Stolk R.P., Glasziou P. (2012). Low HDL Cholesterol and the Risk of Diabetic Nephropathy and Retinopathy. Diabetes Care.

[140] Madsen C.M., Varbo A., Nordestgaard B.G. (2017). Extreme high high-density lipoprotein cholesterol is paradoxically associated with high mortality in men and women: Two prospective cohort studies. Eur. Heart J..

[141] Allard-Ratick M., Khambhati J., Topel M., Sandesara P., Sperling L., Quyyumi A. (2018). 50Elevated HDL-C is associated with adverse cardiovascular outcomes. Eur. Hear. J..

[142] Hirata A., Sugiyama D., Watanabe M., Kotani K., Ueshima H., Imai Y., Ohkubo T., Irie F., Iso H., Kitamura A. (2018). Association of extremely high levels of high-density lipoprotein cholesterol with cardiovascular mortality in a pooled analysis of 9 cohort studies including 43,407 individuals: The EPOCH–JAPAN study. J. Clin. Lipidol..

[143] Ko D.T., Alter D.A., Guo H., Koh M., Lau G., Austin P.C., Booth G.L., Hogg W., Jackevicius C.A., Lee D.S. (2016). High-Density Lipoprotein Cholesterol and Cause-Specific Mortality in Individuals Without Previous Cardiovascular Conditions. J. Am. Coll. Cardiol..

[144] Sobrin L., Chong Y.H., Fan Q., Gan A., Stanwyck L.K., Kaidonis G., Craig J.E., Kim J., Liao W.-L., Huang Y.-C. (2017). Genetically Determined Plasma Lipid Levels and Risk of Diabetic Retinopathy: A Mendelian Randomization Study. Diabetes.

[145] Sasongko M.B., Wong T.Y., Nguyen T.T., Kawasaki R., Jenkins A.J., Shaw J., Wang J.J. (2011). Serum Apolipoprotein AI and B Are Stronger Biomarkers of Diabetic Retinopathy Than Traditional Lipids. Diabetes Care.

[146] Simó R., Garcia-Ramírez M., Higuera M., Hernández C. (2009). Apolipoprotein A1 Is Overexpressed in the Retina of Diabetic Patients. Am. J. Ophthalmol..

[147] Davidson M.H. (2009). Apolipoprotein Measurements: Is More Widespread Use Clinically Indicated?. Clin. Cardiol..

[148] Walldius G., Jungner I. (2006). The apoB/apoA-I ratio: A strong, new risk factor for cardiovascular disease and a target for lipid-lowering therapy—A review of the evidence. J. Intern. Med..

